# Investigation of the Mechanical Performance and Damage Mechanisms of Hybrid Composite Tubes with Mixed Stacking Sequences

**DOI:** 10.3390/ma19143006

**Published:** 2026-07-13

**Authors:** Ayhan Etyemez

**Affiliations:** Department of Mechanical Engineering, Faculty of Technology, Marmara University, Istanbul 34413, Turkey; ayhan.etyemez@marmara.edu.tr; Tel.: +90-5323118994

**Keywords:** hybrid composite tubes, stacking sequence, filament winding, damage mechanisms, radial compression, three-point bending, SEM

## Abstract

In this study, the effect of stacking sequence on the mechanical behavior and damage evolution of carbon- and glass-fiber-reinforced hybrid composite tubes manufactured by the filament winding method was experimentally investigated. The tubes were produced from E-glass and carbon fibers in a Huntsman epoxy matrix, with an inner diameter of 22.5 mm and an outer diameter of 28.5 mm, in two configurations that differ in both the fiber placed in each layer and its winding angle: an inner [±45]_2_ carbon layer/outer [±75]_2_ glass layer (C45) and an inner [±45]_2_ glass layer/outer [±75]_2_ carbon layer (G45). The fiber volume fractions ranged from 0.53 to 0.61 and the measured densities from 1.68 to 1.84 g/cm^3^. Five specimens per configuration (n = 5) were tested under radial compression and three-point bending, and their fracture mechanisms were characterized by scanning electron microscopy (SEM) and optical microscopy. Energy absorption was quantified through the specific energy absorption (SEA). Under radial compression, the C45 configuration exhibited a higher peak force (4209 ± 322 N versus 3573 ± 136 N) and a higher SEA (2.64 ± 0.13 J/g versus 2.06 ± 0.03 J/g). Under three-point bending, C45 again reached a higher flexural strength (92.8 ± 4.5 MPa versus 85.1 ± 3.0 MPa); however, despite failing at a markedly lower peak force, the G45 configuration absorbed a comparable total energy (66.96 ± 2.83 J versus 62.88 ± 6.26 J) and reached a comparable SEA (1.13 ± 0.06 J/g versus 1.08 ± 0.14 J/g), reflecting the ductility-driven damage tolerance imparted by the glass inner layer. Across all metrics, the G45 configuration displayed a consistently lower coefficient of variation (e.g., 3.82% versus 7.64% for radial peak force), indicating greater reproducibility and structural predictability. SEM observations revealed that the C45 specimens failed through sudden fiber fracture and delamination driven by the high stiffness mismatch, indicating brittle behavior, whereas the G45 specimens exhibited progressive damage through matrix crushing and fiber pull-out. The findings indicate that the C45 configuration is favorable where maximum load-bearing capacity and stiffness are targeted, whereas the G45 stacking sequence is advantageous where energy absorption, damage tolerance, and predictable progressive failure are critical.

## 1. Introduction

Today, in advanced engineering disciplines such as aerospace, automotive, marine, and railway systems, the use of lightweight structural materials is of strategic importance to improve fuel efficiency and reduce the carbon footprint. In this regard, conventional metallic alloys are being replaced by fiber-reinforced polymer (FRP) composites, which offer superior specific strength and stiffness characteristics [1,2,3]. The filament winding technique, which enables high automation capability, low production cost, and high fiber volume fractions (Vf), is particularly prominent in the production of tubular structures, pressure vessels, and energy-absorbing components [4,5,6]. Unlike their metallic counterparts, composite structures dissipate energy under axial or lateral loading not through elastoplastic deformation, but through progressive crushing mechanisms such as fiber fracture, matrix cracking, and delamination [7,8,9].

In conventional designs, a single type of fiber, either carbon or glass, is generally used. While carbon fiber-reinforced polymers (CFRP) provide exceptional stiffness, they exhibit brittle fracture behavior due to their low elongation at break; glass fiber-reinforced polymers (GFRP), on the other hand, impart toughness to the structure through their high elongation capability [5,7,9]. To eliminate the disadvantages of these two materials and establish an optimum performance-to-cost balance, hybrid composite structures have been developed in which different fibers are combined within the same matrix. Although the hybridization strategy enhances the impact resistance and damage tolerance of anisotropic structures through a synergistic effect, the stiffness mismatch between the fibers makes it more difficult to predict damage modes [8,9].

There is extensive research in the literature on the mechanical behavior of hybrid tubes. Eggers et al. [10] showed that the winding angle directly affects the radial compression performance of composite tubes and determines their failure modes, while Stabla et al. [11] examined the critical role of different winding angles, such as [±45] and [±75] in the radial compression behavior and energy absorption capacity of CFRP tubes. Cui et al. [6] investigated the crushing characteristics of carbon/glass hybrid tubes and found that the [±45] angle provides excellent performance in terms of energy dissipation. In addition, Zhang et al. [7] demonstrated that design parameters and stacking sequences have a direct and critical effect on Specific Energy Absorption (SEA) in improving the energy absorption capacities of hybrid composite tubes. Gemi [12] emphasized that progressive damage mechanisms in hybrid composite tubes with a glass/carbon/glass sequence progress in the form of delamination, matrix cracks, and fiber damage, while Ozbek [13] examined how interply hybridization differs from intraply hybridization in terms of damage management and thickness uniformity. Furthermore, previous studies [14,15] have emphasized the direct effect of stacking sequence on damage tolerance, while another study [6,9,13] showed that the winding angle determines mechanical stability. Demir et al. [16] found that the use of carbon or glass in the outer layers creates significant differences in frictional energy and collapse characteristics. However, the correlation of macro-mechanical failure criteria such as Tsai–Wu and Tsai–Hill, which are used to predict damage initiation in these complex structures, with experimental data remains a critical research area in the literature. The bending and compression properties of composite structures are strongly dependent on the hybridization technique used, whether interply or intraply. According to fundamental composite mechanics, the overall flexural and compressive stiffness of the structure is directly determined by positioning the high-stiffness carbon fibers at optimum winding angles, while the appropriate stacking sequence of ductile glass fibers significantly improves the energy absorption capacity [17]. In the present study, the interply hybridization technique was preferred because it provides thickness uniformity when fibers with different diameters are used and increases energy dissipation capacity through controlled delamination [18,19,20,21]. Despite this substantial body of work, three specific issues remain unresolved. First, existing studies typically characterize hybrid tubes under a single loading mode, predominantly axial crushing for crashworthiness [6,7,22] or torsion and internal pressure [12,23], so that the radial compression and flexural responses of the same configuration are rarely evaluated together, even though tubular components in service experience both. Second, the influence of fiber stacking position has mostly been examined by varying the winding angle [10,11] or the outer-layer fiber alone [16], whereas the effect of interchanging carbon and glass between the inner and outer layers, while keeping the overall layup architecture fixed, has not been systematically compared. Third, macroscopic indices such as peak load and specific energy absorption are seldom linked directly to the governing microscale damage mechanisms, leaving the structure and property relationship of these hybrids only partially established. The present study addresses these gaps by experimentally comparing two interply hybrid configurations, C45 (inner [±45]_2_ carbon/outer [±75]_2_ glass) and G45 (inner [±45]_2_ glass/outer [±75]_2_ carbon), under both quasi-static radial compression and three-point bending, and by correlating the measured strength, specific energy absorption, and variability directly with the SEM- and optical-microscopy-based damage mechanisms. Unlike prior single-load or single-mechanism studies, this combined macro–micro approach isolates the role of inner vs. outer fiber placement on both load-bearing capacity and damage tolerance within a single hybrid system. By targeting a demanding and widely used structural application composite drive shafts, which operate under combined radial compression and bending loads and demand both high stiffness and reliable damage tolerance, this study extends the design principles of hybrid composite tubes beyond those established for conventional metallic and single-fiber shafts [24,25,26,27]. Within the scope of the study, the effect of fiber stacking sequence on structural performance was tested in two different hybrid tube configurations, with an average inner diameter of 22.5 mm and outer diameter of 28.5 mm, manufactured with an inner-layer winding angle of [±45]_2_ and an outer-layer winding angle of [±75]_2_: C45, with a carbon inner layer, and G45, with a glass inner layer. When the main findings of the study are examined, the C45 configuration, in which carbon is wound at the inner [±45]_2_ orientation, exhibited the highest radial compression force (4209 N) and three-point bending force (5161 N), thereby providing the greatest structural stiffness. In contrast, the G45 configuration, in which glass occupies the inner [±45]_2_ orientation, provided much higher damage tolerance and statistical predictability, attributed to the ductile nature of the glass fiber together with matrix crushing and progressive damage mechanisms. By integrating the obtained data with macroscopic mechanical tests, Scanning Electron Microscopy (SEM) characterization, and theoretical damage approaches, this study provides the literature with a comprehensive and original guide for multiple loading conditions encountered in the design of industrial lightweight structures.

## 2. Materials and Methods

In this study, E-glass fibers (E-glass fibers (600 tex, Şişecam, Istanbul, Turkey) and carbon fibers (12K A-42, DowAksa, Yalova, Turkey) were used as reinforcement materials in the production of cylindrical hybrid composite tubes. As the matrix material, a thermoset epoxy resin mixture of Huntsman My740—HY918—DY062 (Huntsman Advanced Materials, Basel, Switzerland) and Epikure 875 (Hexion Inc., Columbus, OH, USA) hardener was selected as mechanical properties shown in Table 1. Within the scope of the study, tubes with an average length of 1.5 m, an inner diameter of 22.5 mm, and an outer diameter of 28.5 mm were designed. To systematically investigate the effects of fiber type and stacking sequence on mechanical performance, two different hybrid configurations were defined:

- Configuration C45 (Carbon Inner Layer): Inner layer: [±45]_2_ carbon fiber/Outer layer: [±75]_2_ glass fiber
- Configuration G45 (Glass Inner Layer): Inner layer: [±45]_2_ glass fiber/Outer layer: [±75]_2_ carbon fiber

The selection of this specific layer arrangement was based on findings in the literature indicating that placing carbon fiber at the inner [±45]_2_ orientation tends to increase the bending and compression stiffness of the structure under local loading, while positioning glass fiber in the inner layer provides advantages in terms of impact resistance and cost optimization by imparting toughness to the structure [28,29].

### 2.1. Filament Winding Process and Curing Conditions

The specimens were manufactured using the wet and helical winding technique with a 4-axis CNC-controlled filament winding machine. During production, the fiber bundles were passed through an epoxy resin bath and placed onto the mandrel at [±45] and [±75] winding angles; thus, an optimum balance between hoop and axial load-bearing capacities was targeted. To improve winding quality and prevent any adverse effect on the viscosity properties of the resin, the mandrel was preheated to 60 °C before the winding process. To ensure process continuity and improve demolding efficiency, a mold release agent was applied to the surface of the mandrel. Throughout the process, the winding tension was kept constant and applied in the range of 8–10 N to ensure complete wetting of the fibers with the matrix and to minimize structural voids. After the winding process was completed, the specimens were cured in a temperature-controlled rotating oven at 150 °C for 4 h to allow the composite tubes to complete the cross-linking reaction.

**Figure 1:**
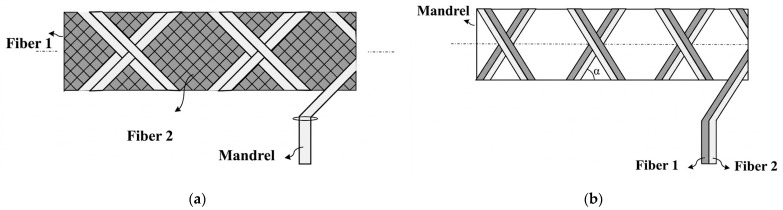
(**a**) Interply winding technique. (**b**) Intraply winding technique [13].

There are two different methods that are widely used in the literature for hybridizing different types of fibers. These are the interply and intraply techniques, respectively [13,18]. In interply fiber hybridization, as shown in Figure 1a, after one layer is completely covered with the first fiber, the next layer is wound over the first layer using a different fiber reinforcement. Therefore, different layers must consist of different fiber reinforcements. In contrast, in intraply fiber hybridization, as shown in Figure 1b, at least two fiber reinforcements are wound together within the same layer, and each layer of the composite tube contains the same material. These techniques have certain advantages and disadvantages compared with each other. While the interply technique requires the filament system to be reconfigured for the continuation of each layer, the process is completed without any intervention in the intraply technique. However, in the intraply technique, the diameters of the different fibers used must be very close to one another to ensure uniform product thickness. In the interply technique, however, this is not required because the different layers are independent of one another [13,18]. In this study, the interply winding technique was preferred instead of the intraply technique as the hybridization strategy in order to eliminate the aforementioned disadvantage related to thickness uniformity. Through the interply technique, carbon and glass fibers with different fiber diameters could be used independently. In addition, by forming distinct interfaces between carbon and glass layers with different stiffness levels, this method aims to slow crack propagation under radial compression and bending loads, to dissipate the applied mechanical energy more effectively through a controlled delamination mechanism, and to improve the overall damage tolerance of the tubes.

### 2.2. Specimen Preparation and Burn-Off Test

After the curing process was completed, the composite tubes were precisely cut using a Metkon abrasive cutting machine (Metkon Instruments Inc., Bursa, Turkey) equipped with a diamond/ceramic cutting disc in order to obtain dimensions suitable for mechanical testing standards. After cutting, wet sanding was applied to the specimen cross-sections using 300- and 800-grit abrasives, respectively, in order to eliminate notch effects (stress concentrations) that could form on the end and edge surfaces and cause premature brittle fractures during testing Within the scope of the mechanical testing program, five specimens from each configuration (C45 and G45) were prepared for the radial compression tests, and five specimens from each configuration were prepared for the three-point bending tests. For the three-point bending specimens of the C45 and G45 configuration tubes, before testing, a burn-off test was applied to the specimens in an ash furnace in accordance with ASTM D2584 in order to determine the net fiber ratios of the manufactured hybrid composite tubes, and the fiber volume fraction (Vf) of each specimen was precisely determined [30].

The physical properties, stacking sequences, densities, and fiber volume fractions obtained from the burn-off test for the manufactured hybrid composite tubes on a specimen-by-specimen basis are presented in detail in Table 2.

The void content of the manufactured tubes was estimated by comparing the experimentally measured densities with the theoretical densities obtained from the rule of mixtures, using the measured fiber volume fractions together with the densities of the carbon fiber, glass fiber, and epoxy matrix. The measured and theoretical densities were in close agreement, indicating a low void content. This finding is consistent with the information provided by the tube manufacturer, according to which the void content remained below 5%, as well as with values reported in the literature for comparable filament-wound glass/epoxy tubes [31,32].

### 2.3. Mechanical Tests

#### 2.3.1. Radial Compression Test

The tubes were prepared as short ring sections with a length of 36 mm, and load-deformation curves were obtained using a standard ring compression fixture in accordance with ASTM D2412 [33]. A total of ten test specimens, five from each of the C45 and G45 configurations, were prepared and tested.

**Figure 2:**
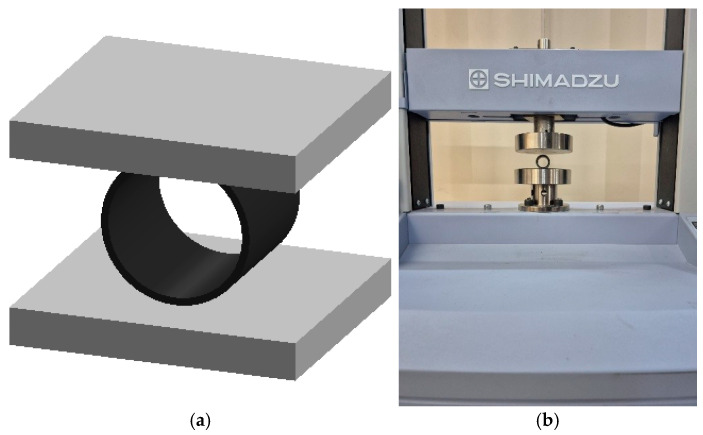
(**a**) technical drawing view of the composite tubes used; (**b**) Shimadzu AGS-X 10kN universal tensile-compression testing machine and loading system for compression test.

As shown in Figure 2, the specimens were subjected to compressive loading between two parallel steel plates to ensure a uniform load distribution in accordance with the ASTM D2412 standard [33]. The test method focuses on the displacement characteristics of composite tubes under parallel-plate loading. Based on these loading characteristics, stiffness (S), percent ring deflection (PRD), and stiffness factor (SF) are calculated using Equation (1), Equation (2), and Equation (3), respectively, as follows [10]:

S = F/∆y(1)

F: Applied force (N), Δy: Displacement (mm)

PRD = (∆y/D)∙100(2)

Δy: Displacement (mm), D: Initial tube diameter (mm)

SF = S∙t(3)

S: Stiffness (N/mm), t: Tube wall thickness (mm).

#### 2.3.2. Three-Point Bending Test

Three-point bending tests were performed to determine the bending behavior and maximum flexural strength of the manufactured hybrid composite tubes. Unlike the short ring sections used in the radial compression test, the axial integrity of the C45 and G45 composite tubes was preserved for the bending test specimens; the tubes were cut and prepared as whole tube sections with a total length of 140 mm, suitable for the 100 mm support span of the testing device. The prepared tube specimens were subjected to bending tests using a Shimadzu AGS-X 10kN (Shimadzu Corporation, Kyoto, Japan) universal testing machine with reference to ASTM D790 [34]. The tests were conducted with a lower support span of 100 mm and a constant crosshead speed of 2 mm/min. Using the three-point bending fixture, the force applied at the exact midpoint of the specimen and the corresponding deflection (displacement) values were simultaneously recorded in a computer environment, and force–displacement curves were obtained for each configuration.

The flexural strength (σ) of the specimens was calculated for the hollow circular cross-section using Equation (4) where F is the maximum force, L is the support span (100 mm), c is the outer radius, and I = π(D_o^4^ − D_i^4^)/64 is the second moment of area of the tube section.

σ = F·L·c/(4I)(4)

**Figure 3:**
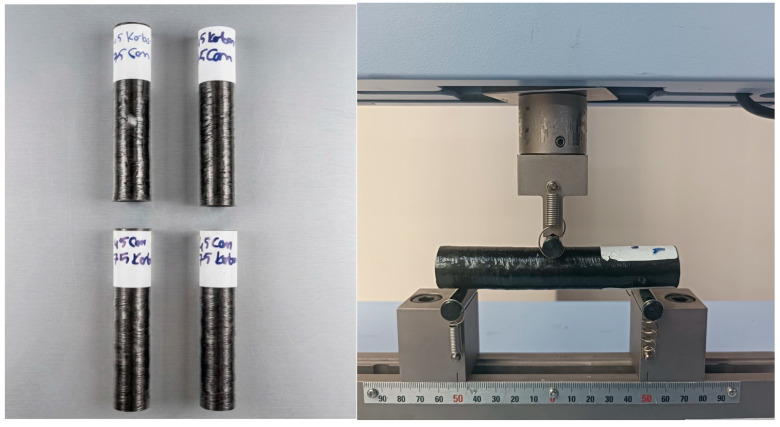
Three-point bending specimens specimens (with handwritten specimen identification labels) and typical deformation views at different strain values.

The flexural behavior and macroscopic damage evolution of the hybrid composite tubes were evaluated through three-point bending tests in accordance with the ASTM D790 standard, and the typical test setup along with the deformation stages are illustrated in Figure 3.

#### 2.3.3. Energy Absorption Analysis

**Figure 4:**
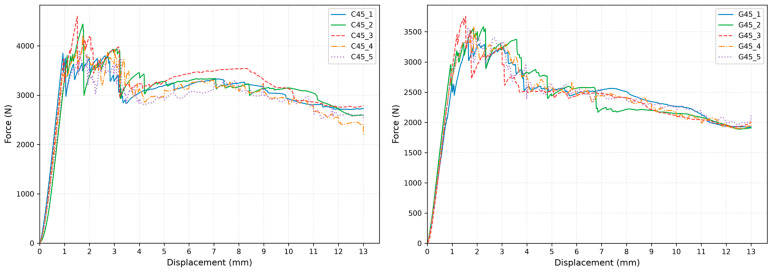
Radial compression force–displacement curves of the individual C45 and G45 specimens.

**Figure 5:**
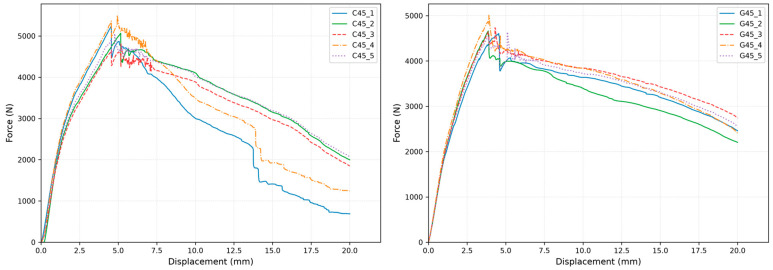
Three-point bending test force–displacement curves of the individual C45 and G45 specimens.

The energy absorption capacity of the hybrid tubes was determined from the experimental force–displacement curves obtained in the radial compression and three-point bending tests (Figure 4 and Figure 5). The absorbed energy (E) was calculated as the area under the force–displacement curve, evaluated by numerical integration up to a fixed displacement for each loading mode—13 mm for radial compression and 20 mm for three-point bending—so that the two configurations were compared over identical deformation ranges, as given in Equation (5).

E = ∫_0_^δ F(x) dx (5)

where F is the instantaneous force (N) and δ is the prescribed integration limit (mm). The specific energy absorption (SEA), which normalizes the absorbed energy with respect to mass, was then obtained from Equation (6)

SEA = E/m(6)

where m is the specimen mass (g), calculated from the measured density and the hollow-cylinder geometry. For the three-point bending tests, the mean crushing load (P_m_) was additionally evaluated as P_m_ = E/δ. To assess the consistency of each configuration, the coefficient of variation (COV), defined as the ratio of the standard deviation to the mean, was reported for all measured quantities. The resulting energy absorption indices for the two loading modes are summarized in Table 3 and Table 4, respectively.

### 2.4. Damage and Fracture Analysis

After the mechanical tests, the fracture regions of the specimens were first evaluated at the macro scale through visual and optical examination, and damage types such as fiber fracture, matrix cracks, fiber pull-out, and interlaminar separation were observed. Sections taken from representative specimens were examined using scanning electron microscopy (SEM), and the microstructural damage mechanisms were classified in detail. This analysis was used to reveal the effect of stacking sequence on damage initiation and propagation.

Detailed imaging techniques were employed to characterize the macroscopic and microscopic damage mechanisms that occurred in the specimens after the radial compression and three-point bending tests. The macro-scale damage and surface cracks in the specimens were first examined using an OLYMPUS SZX7 (Olympus Corporation, Tokyo, Japan) high-resolution microscope camera system. To identify microstructural damage requiring more detailed examination, sections were carefully taken from the main damage regions visually detected after the mechanical tests, and the specimens were prepared for microscopic analysis. Complex failure modes in these prepared specimens, such as fiber-matrix interfacial debonding, delamination, micro-matrix cracks, and fiber fractures, were analyzed in detail at high magnifications using an FEI QUANTA FEG 250 (FEI Company, Hillsboro, OR, USA) scanning electron microscope (SEM).

## 3. Results

**Figure 6:**
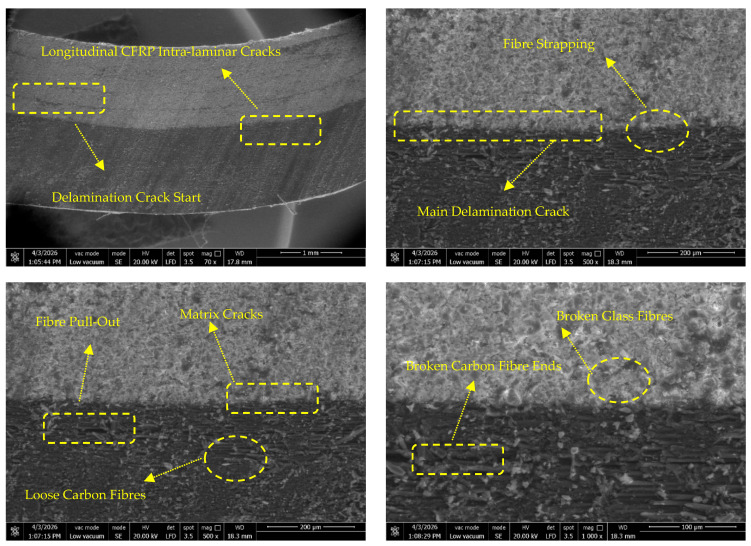
SEM Images of G45 Specimens After Radial Compression.

**Figure 7:**
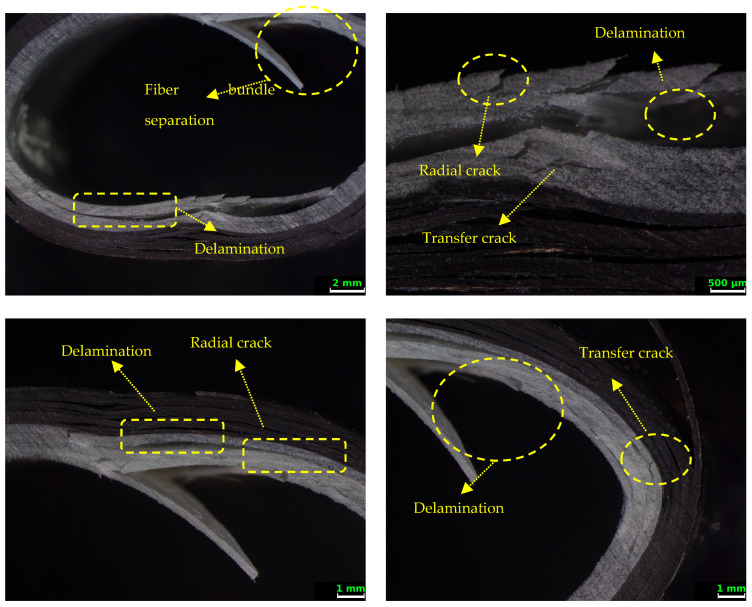
Optical Microscope Images of G45 Specimens After Radial Compression.

**Figure 8:**
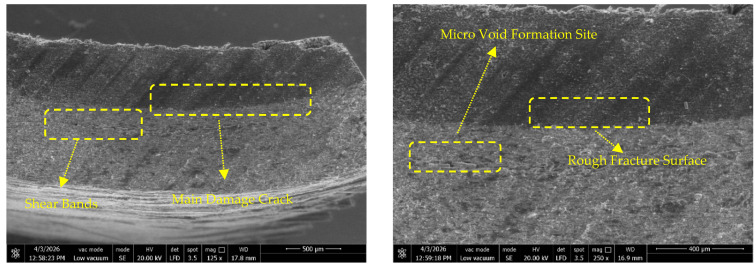

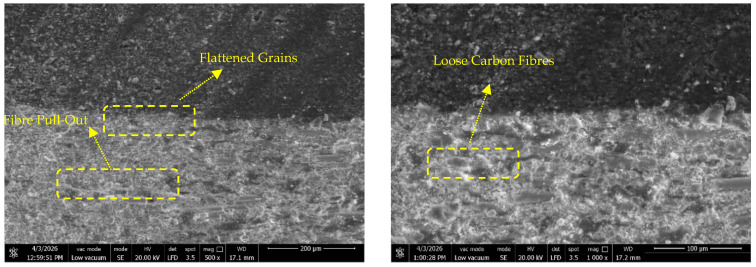
SEM Images of C45 Specimens After Radial Compression.

**Figure 9:**
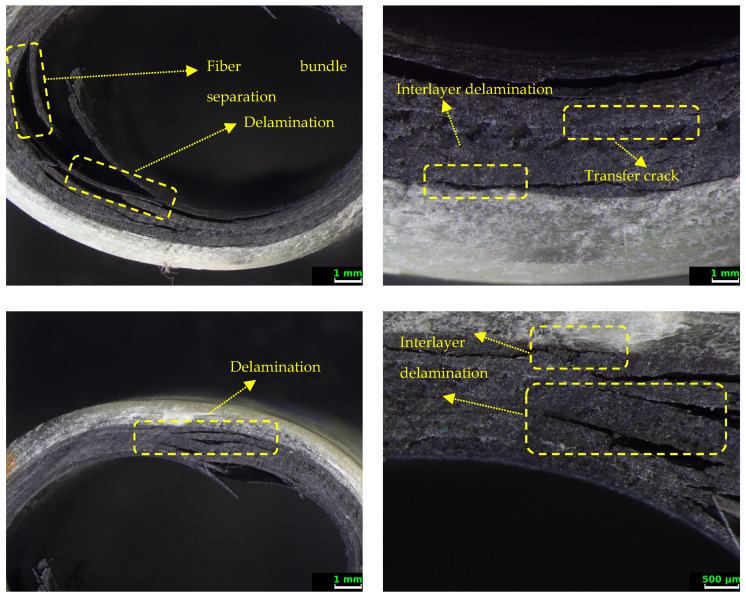
Optical Microscope Images of C45 Specimens After Radial Compression.

The macroscopic test results and statistical data directly correspond to the microscopic damage mechanisms obtained from the SEM analyses. Although the C45 configuration, in which carbon is wound at the inner [±45]_2_ orientation, reached the highest average peak force in the radial compression tests (4209 N), its higher coefficient of variation (COV) of 7.64% indicates the more brittle nature of the material. Damage modes such as fiber–matrix interfacial debonding and matrix crushing observed in the SEM and optical microscopy analyses of the G45 configuration (Figure 6 and Figure 7) demonstrate that the structure did not fail suddenly; rather, it gradually absorbed the applied load and energy through micro-deformations. This demonstrates, through both statistical consistency and microstructural findings, the damage tolerance capability imparted to the composite tube by the use of glass fiber in the inner layer. The sudden fiber fractures (brittle fiber fracture) and extensive delaminations resulting from the stiffness mismatch detected in the SEM and optical microscopy images of the C45 specimens (Figure 8 and Figure 9) confirm that the structure experienced sudden load drops without dissipating energy, thereby increasing the performance variability among the specimens, namely the standard deviation [35]. In contrast, the statistically much more consistent and predictable behavior of the G45 configuration, in which glass occupies the inner [±45]_2_ orientation (radial compression COV = 3.82%), is explained by the high ductility and deformation capability of glass fiber [36,37]. 

### 3.1. Load-Deformation Behavior

#### 3.1.1. Radial Compression Test Analysis

As a result of the radial compression tests, the typical force–displacement curves of the C45 and G45 configurations presented in Figure 4 were obtained, and the corresponding energy absorption indices are summarized in Table 3. When the mechanical responses and the maximum load-bearing capacities are examined, it is observed that the C45 configuration, in which carbon is wound at the inner [±45]_2_ orientation, reached an average maximum compression force of 4209 N, approximately 17.8% higher than that of the G45 configuration, in which glass occupies the inner [±45]_2_ orientation (average 3573 N). The C45 configuration also exhibited a higher specific energy absorption (2.64 J/g versus 2.06 J/g for G45). This can be attributed to positioning the carbon fibers with a high elastic modulus at the inner [±45]_2_ orientation, which best accommodates the multiaxial stresses generated under radial loading and thereby maximizes the structural stiffness and load-bearing capacity of the tube [38,39]. As also reported in studies by Eggers et al. [10] and Stabla et al. [11], positioning high-stiffness fibers at appropriate winding angles directly improves performance under lateral compression.

However, when the damage progression behavior after the peak point in the force–displacement curves was examined, it was determined that the G45 specimens released the load more gradually and exhibited a more stable deformation response, indicating ductile behavior. The G45 configuration exhibited high repeatability, with a low coefficient of variation (COV) of 3.82% in peak force. This stable behavior demonstrates that, due to the high elongation at break of the glass fiber in the inner layer, matrix crushing and progressive damage development increase the damage tolerance of the material [40]. In contrast, the higher coefficient of variation of 7.64% in the C45 specimens, together with the sudden load drops observed in the curves in Figure 4, can be attributed to sudden fiber fractures and local delaminations caused by the brittle nature of carbon fiber, which abruptly disrupt structural integrity [40,41]. These results indicate that the C45 configuration is advantageous in designs requiring maximum static load-bearing capacity, whereas the G45 configuration is advantageous in cases requiring predictable damage progression and consistent energy absorption.

#### 3.1.2. Three-Point Bending Test Analysis

When the force–displacement curves obtained from the three-point bending tests (Figure 5) and the maximum force distributions (Table 4) are examined, it is once again seen that the stacking sequence plays a decisive role in the bending behavior of hybrid composite tubes. According to the test results, the C45 configuration, in which carbon is wound at the inner [±45]_2_ orientation, exhibited the highest flexural strength, reaching an average maximum force of 5161 N (corresponding to a flexural strength of 92.8 MPa) and an elastic-region stiffness of 742 N/mm. In contrast, the G45 configuration, in which glass occupies the inner [±45]_2_ orientation, had an average maximum force of 4731 N (85.1 MPa) and a stiffness of 607 N/mm. This corresponds to an approximately 9.1% higher peak force for the C45 configuration. This indicates that, in the stress distribution to which the structure is subjected during bending, positioning the high-elastic-modulus carbon fiber at the inner [±45]_2_ orientation increases bending resistance [42].

When evaluated in terms of damage mechanisms and deformation behavior, the G45 configuration was found to exhibit a much more stable profile. The G45 specimens exhibited high repeatability, with a low coefficient of variation (COV) of 3.47% in maximum force, whereas this value was 4.83% for the C45 specimens. Moreover, despite failing at a lower peak force, the G45 configuration absorbed a comparable total energy (66.96 J versus 62.88 J) and reached a comparable specific energy absorption (1.13 J/g versus 1.08 J/g), reflecting the ductility-driven damage tolerance imparted by the glass inner layer. When the post-peak decline trends of the curves are examined (Figure 5), sudden load drops caused by the brittle nature of carbon fiber are observed in the C45 configuration [3,41,42]. In contrast, in the G45 configuration, which has a glass inner layer, ductile deformation was maintained up to the fracture point due to the high elongation capability of glass fibers, and the displacement progressed more uniformly [4,40]. These findings confirm that the C45 configuration is advantageous when flexural strength and stiffness must be maximized, whereas the G45 configuration is advantageous when structural predictability and damage tolerance are critical.

### 3.2. Microstructural Damage and Fracture Analyses

**Figure 10:**
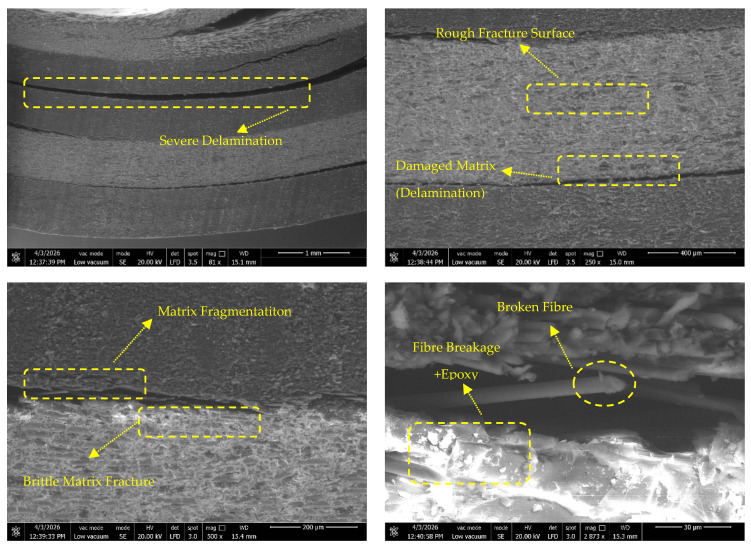
SEM Images of G45 Specimens After Three-Point Bending.

**Figure 11:**
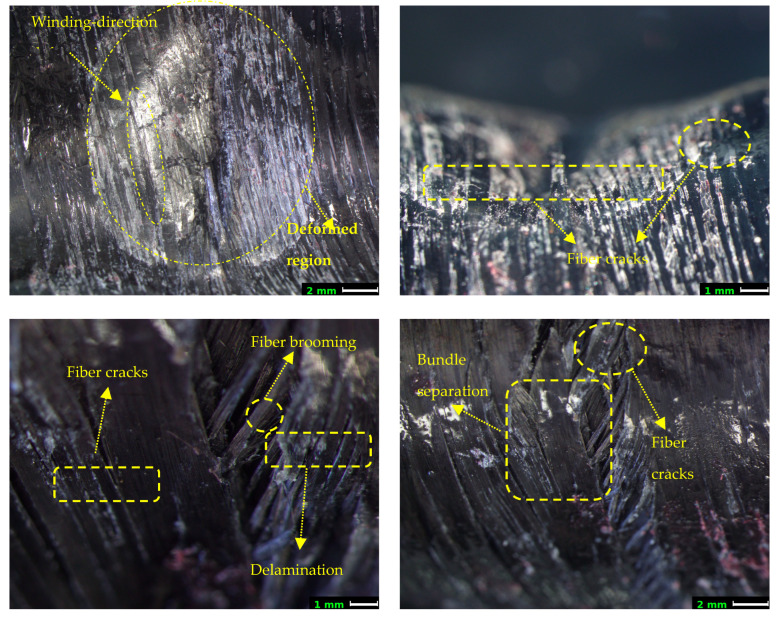
Optical Microscope Images of G45 Specimens After Three-Point Bending.

**Figure 12:**
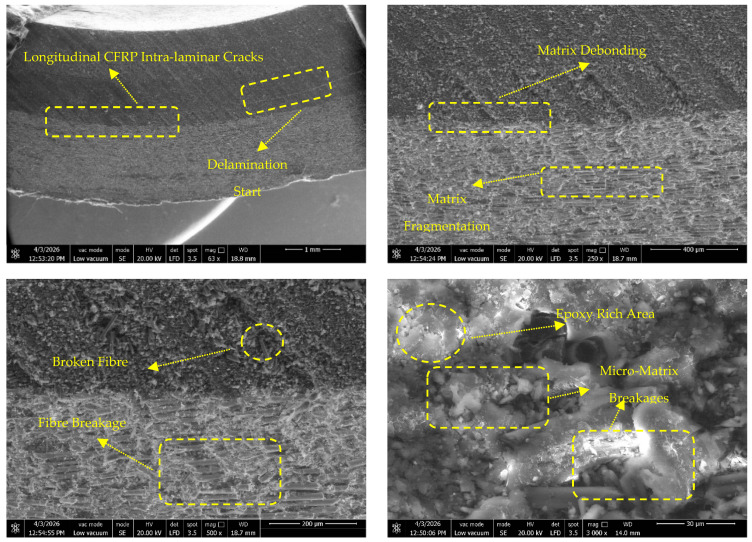
SEM Images of C45 Specimens After Three-Point Bending.

**Figure 13:**
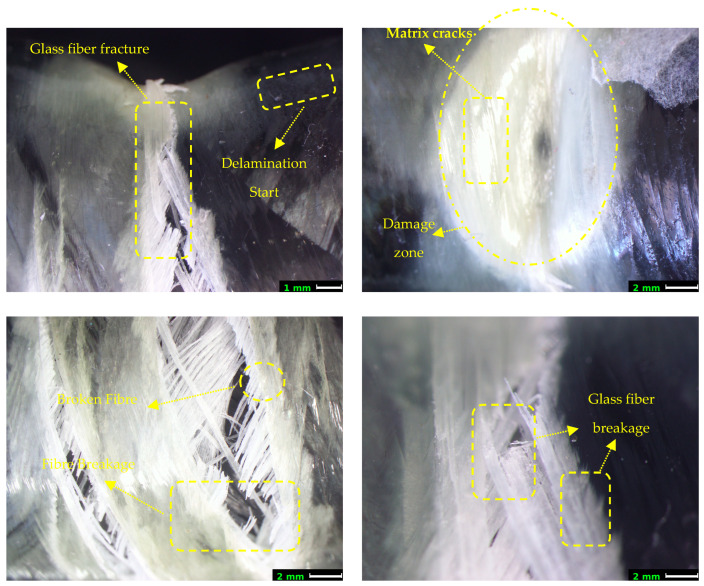
Optical Microscope Images of C45 Specimens After Three-Point Bending.

Macro-scale examinations and SEM analyses (Figure 6, Figure 7, Figure 8, Figure 9, Figure 10, Figure 11, Figure 12 and Figure 13) revealed that matrix cracking, local buckling, fiber fracture, and interlaminar separation (delamination) were the dominant damage modes in both configurations [43]. In the C45 configuration, sudden load drops were more dominant as a result of the brittle fracture of the carbon fibers located in the inner layer. In contrast, in the G45 configuration, more widespread fiber pull-out in the glass inner layer and progressive crack propagation in the matrix were observed, contributing to its more ductile and damage-tolerant behavior [44,45]. The SEM images showed that fiber fracture (brittle breakage) was dominant in the layers containing carbon fiber rather than failure at the fiber/matrix interface. In the glass fiber layers, however, fiber pull-out, interfacial debonding, and matrix bridging were much more pronounced. As theoretically predicted by macroscopic failure criteria (e.g., Tsai–Wu), these microscopic findings confirm that the severe stiffness mismatch between the carbon and glass layers leads to high interlaminar shear stresses. These stresses trigger delamination and micro-matrix cracks at the interfaces, ultimately determining the overall damage progression and energy absorption capacity of the hybrid composite tube [44,45].

#### 3.2.1. Radial Compression Images

When the SEM (Figure 6) and optical microscope (Figure 7) images of the G45 configuration are examined, progressive damage mechanisms resulting from the ductile nature of the inner glass fibers are clearly observed. Microstructural defects such as “Fibre Pull-Out” and “Matrix Cracks” indicate that the applied mechanical energy is gradually absorbed by the structure through extensive matrix crushing rather than causing sudden catastrophic failure. Although a main delamination crack is detected at the interfaces, the high elongation capability of the inner glass layers accommodates large deformations, maintaining structural integrity up to higher strain levels. This progressive microstructural deterioration is consistent with the gradual post-peak load decay and the stable, low-variability energy absorption observed in the macroscopic tests, where the G45 configuration sustained a comparable energy absorption to C45 despite its markedly lower peak load.

In contrast, the microstructural damage profile of the C45 configuration exhibits a distinctly brittle fracture characteristic. While the high elastic modulus of the inner carbon layer provides superior stiffness and peak load capacity, it inevitably leads to catastrophic failure once the ultimate stress limit is reached. Damage in this configuration developed in a rapid, brittle sequence: matrix microcracking first initiated within the inner carbon layer, which was almost immediately followed by sudden fiber fracture near the peak load and by rapid “Interlayer Delamination” driven by the severe stiffness mismatch between the inner carbon and outer glass layers. The presence of “Loose Carbon Fibres”, “Flattened Grains”, and this rapid interlayer delamination observed in the SEM images is a direct consequence of this mismatch. Because these stages occurred over a very narrow displacement range rather than progressively, the sudden and massive fiber breakages severely limited the extended energy dissipation capability of the tube, microstructurally confirming the sudden load drops and the higher variability in fracture behavior observed in the force–displacement curves.

#### 3.2.2. Three-Point Bending Images

Under three-point bending loads, the failure evolution of the G45 configuration is predominantly characterized by “Severe Delamination” and “Matrix Fragmentation,” as shown in Figure 10 and Figure 11. The bending forces, which induce simultaneous tensile and compressive stresses, are initially accommodated by the ductile inner glass layer; however, the stress transfer to the outer carbon layer results in a “Rough Fracture Surface” and “Brittle Matrix Fracture” at the interface. The “Winding-direction deformation” observed in the layers suggests that while the structure attempts to flex under the bending load, the resin matrix reaches its ultimate strain limit, leading to localized fragmentation. Nevertheless, the absence of massive fiber rupture in the core confirms the excellent statistical repeatability and stable post-peak behavior of the G45 tubes.

Figure 12 and Figure 13 illustrate the complex damage mechanisms of the C45 specimens under flexural loading, where “Fibre Breakage” and “Micro-Matrix Breakages” are highly pronounced. The failure is heavily concentrated around “Epoxy Rich Areas,” where “Matrix Debonding” and “Longitudinal CFRP Intra-laminar Cracks” rapidly propagate through the weakest interfaces. As the configuration with the highest bending stiffness, the C45 tube withstands significant loads until the tensile stresses at the lower surface exceed the failure threshold of the carbon fibers. At this critical point, mechanisms such as “Fiber brooming” and massive “Bundle separation” occur simultaneously, leading to abrupt brittle fracture. The extensive glass fiber fracture and delamination initiation zones in Figure 13 highlight how the initial carbon fiber failure triggers a chain reaction of catastrophic damage across the entire hybrid cross-section. In terms of damage evolution, the two configurations followed distinctly different sequences, consistent with the damage progression reported for filament-wound and hybrid composite tubes in the literature, where matrix cracking is the earliest and lowest-energy mechanism, followed by fiber–matrix debonding and delamination, with fiber breakage occurring last, typically near the peak load [46]. In the C45 configuration, damage initiated as matrix microcracking within the inner carbon [±45]_2_ layer; as the load approached its peak, this was followed by abrupt fiber fracture and rapid interlaminar delamination driven by the stiffness mismatch at the carbon–glass interface, culminating in a sudden loss of load-bearing capacity. In the G45 configuration, damage also initiated as matrix cracking in the inner glass layer, but instead of abrupt fiber failure, it progressed through fiber–matrix debonding, fiber pull-out, and gradual matrix crushing; the high strain capability of the glass fibers delayed final fiber breakage and produced the gradual, stable load decay reflected in the lower coefficient of variation [47].

### 3.3. Analytical Prediction of Damage Initiation (Tsai–Wu Criterion)

To provide a theoretical basis for the experimentally observed damage-initiation behaviour, a first-ply failure analysis based on the Tsai–Wu criterion was carried out. The analysis follows a closed-form analytical approach based on classical lamination theory (CLT); no finite-element model was employed. The three-point bending configuration was selected for this exercise because, unlike the ring-compression test, it produces a well-defined in-plane stress state that can be evaluated analytically. The stress state was taken directly from the experiments: the measured average peak force of each configuration (5161 N for C45 and 4731 N for G45) was converted into the outer-surface axial stress of the tube using the hollow-section flexural relation already given in Equation (4), yielding 92.8 MPa for C45 and 85.1 MPa for G45. In this way the analytical prediction is evaluated under the same geometry (Do = 28.5 mm, Di = 22.5 mm, span = 100 mm) and the same load level as the physical tests.

The lamina elastic constants of the inner [±45]_2_ load-bearing layer were derived from the constituent fiber and matrix properties given in Table 1 using micromechanics: the axial modulus E1 and major Poisson ratio ν_12_ from the rule of mixtures, and the transverse modulus E2 and in-plane shear modulus G_12_ from the Halpin–Tsai equations (ξ = 2 and ξ = 1, respectively) [48,49]. The resulting elastic constants and the corresponding lamina strengths are listed in Table 5 and Table 6. The longitudinal strengths were obtained from the rule of mixtures, the transverse and shear strengths were taken from the manufacturer-measured values of the glass/epoxy system and consistent literature values for the carbon/epoxy system, and the compressive strengths were estimated from established literature ratios [49,50]. Using CLT, the outer-surface axial stress was transformed into the principal material axes (1,2) of the inner layer. Importantly, the [±45]_2_ layer was not treated as a single equivalent ply: the +45° and −45° plies were evaluated separately, and the Tsai–Wu criterion was applied ply-by-ply to each of them. The criterion [50,51] has the form given in Equation (7):

(7) $$F_{1}\sigma_{11}+F_{2}\sigma_{22}+F_{6}\tau_{12}+F_{11}\sigma_{11}^{2}+F_{22}\sigma_{22}^{2}+F_{66}\tau_{12}^{2}+2F_{12}\sigma_{11}\sigma_{22}=1$$

(8) $$F_{1}=\frac{1}{S_{Lt}}-\frac{1}{S_{Lc}},F_{2}=\frac{1}{S_{Tt}}-\frac{1}{S_{Tc}},F_{6}=0$$

(9) $$F_{11}=\frac{1}{S_{Lt}S_{Lc}},F_{12}=-\frac{1}{2S_{Lt}^{2}},F_{22}=\frac{1}{S_{Tt}S_{Tc}},F_{66}=\frac{1}{S_{LTs}^{2}}.$$

where the coefficients F_i_ and F_ij_ (linear and quadratic coefficients) are defined in terms of the lamina strengths (Equations (8) and (9)). Because the criterion is quadratic in the applied stress, its evaluation gives a strength ratio (SR) for each ply. The strength ratio is defined as the factor by which the applied stress state can be scaled before the Tsai–Wu criterion reaches unity (first-ply failure). A value of SR = 1 corresponds to the onset of damage, SR > 1 indicates a remaining margin against failure, and a lower SR therefore denotes earlier damage initiation. For each configuration the +45° and −45° plies gave identical strength ratios, since the shear term enters the criterion quadratically; the reported SR is therefore the critical (governing) value of the inner layer. The reliability of the derived constants was verified in two ways. First, the predicted glass/epoxy transverse tensile strength (≈51 MPa) and shear strength (≈78 MPa) agree closely with the values measured on the same Huntsman MY740/E-glass system (48–55 MPa and 77–80 MPa, respectively). Second, the derived elastic constants (E1 = 40.7 GPa, G12 = 3.9 GPa for glass/epoxy) are essentially identical to widely used reference values for unidirectional E-glass/epoxy at a comparable fiber volume fraction [49]. These agreements confirm that the micromechanical inputs are appropriate for the present material system. Under the experimental peak-load stress state, the Tsai–Wu evaluation gives a lower strength ratio for the inner [±45]_2_ carbon layer of C45 (SR = 0.82) than for the inner [±45]_2_ glass layer of G45 (SR = 0.95), i.e., the carbon inner layer of C45 reaches the failure envelope earlier than the glass inner layer of G45 (Table 7). This relative ordering is fully consistent with the experimental results: the earlier damage initiation predicted for the inner carbon layer corresponds to the sudden load drops and the higher coefficient of variation measured for C45, and to the brittle fiber fracture and delamination identified in the SEM analyses. Conversely, the larger strength ratio of the ductile inner glass layer in G45 is consistent with its progressive, stable post-peak load decay and lower variability. It should be emphasised that this analysis is intended as a relative, first-ply comparison between the two inner layers rather than an absolute strength prediction; a full progressive-damage finite-element simulation, accounting for the complete ply sequence and the multiaxial ring-compression stress field, is identified as a direction for future work.

## 4. Discussion

The macroscopic data obtained from the radial compression and three-point bending tests, together with the Scanning Electron Microscopy (SEM)-based microstructural analyses, show that the stacking sequence of carbon and glass fibers in the interply hybridization technique directly determines the mechanical performance, failure behavior, and damage tolerance of composite tubes. The C45 configuration, in which carbon is wound at the inner [±45]_2_ orientation, achieved the highest peak load and stiffness under both radial compression (average 4209 N) and three-point bending (average 5161 N). Positioning the high-elastic-modulus carbon fiber at the inner [±45]_2_ orientation, which better accommodates the multiaxial stresses generated under these loadings, maximized the overall load-bearing capacity of the structure. In the G45 configuration, by contrast, the carbon fiber is located in the outer layer at a [±75]_2_, hoop-dominated orientation, so its contribution to the load-bearing capacity under radial compression and bending remained more limited. However, despite its higher peak load, the higher coefficient of variation (COV) of the C45 specimens (7.64% in compression and 4.83% in bending), together with the sudden fiber fractures and severe delaminations detected in the SEM images, indicates that the structure has a more brittle fracture characteristic. In contrast, although the G45 configuration, which has a glass fiber inner layer, remained behind C45 in terms of maximum strength, it exhibited excellent deformation stability and damage tolerance The lower coefficients of variation exhibited by the G45 specimens (3.82% in compression and 3.47% in bending) demonstrate the greater predictability of the structure against damage. As also confirmed by the SEM analyses, due to the high elongation at break of glass fiber, mechanisms such as fiber-matrix interfacial debonding, matrix crushing, and fiber pull-out occurred in the specimens instead of sudden fractures. This ductile behavior, which slows crack propagation, enabled the structure to gradually dissipate the applied load and impact energy. These findings are fully theoretically consistent with the results reported by Cui et al. and Zhang et al. in their studies on hybrid tubes, which indicated that the use of glass fiber increases structural ductility [6,7]. The obtained results show that the stacking sequence of hybrid tubes in industrial lightweight structural designs should be optimized according to the intended application. The use of the C45 configuration, with carbon wound at the inner [±45]_2_ orientation, is advantageous in structural components where absolute stiffness and high load-bearing capacity are targeted. Conversely, the G45 stacking sequence is advantageous in cases where energy absorption, damage tolerance, and predictable progressive failure are critical, owing to the ductile nature of the glass inner layer.

## 5. Limitations

Several limitations of the present study should be acknowledged. First, the C45 and G45 configurations differ simultaneously in two respects: the type of fiber placed in the inner versus the outer layer, and the winding angle at which each fiber is oriented, since carbon and glass occupy the [±45]_2_ and [±75]_2_ orientations interchangeably between the two configurations. Consequently, the observed performance differences reflect the combined effect of fiber type, stacking position, and winding angle, and cannot be attributed solely to the presence of carbon or glass in the inner layer; isolating these individual contributions would require additional configurations in which fiber type and winding angle are varied independently. Second, five specimens were tested per configuration for each loading mode; although this sample size is sufficient to reveal consistent trends and to quantify variability through the coefficient of variation, larger sample sizes would further strengthen the statistical robustness of the comparisons, and the descriptive statistics reported here (mean ± standard deviation and coefficient of variation) were therefore preferred over formal parametric significance testing. Third, the void content was estimated indirectly from density measurements, which quantify the total porosity; a direct quantification of open porosity by water-absorption (Archimedes) or mercury-intrusion porosimetry, together with a dedicated microscopy survey of pristine-state resin pockets and fiber–matrix debonding, was not performed and would further characterize the manufacturing quality. These aspects are identified as directions for future work.

## 6. Conclusions

In this study, the mechanical performance and microstructural damage mechanisms of carbon- and glass-fiber-reinforced hybrid composite tubes manufactured by the filament winding method, namely the C45 and G45 configurations, were experimentally investigated under radial compression and three-point bending loading. The test results show that fiber stacking sequence is decisive for structural integrity in the interply hybridization strategy. In the radial compression tests, the C45 configuration, in which carbon is wound at the inner [±45]_2_ orientation, reached an average force of 4209 N, approximately 17.8% higher than the G45 configuration, in which glass occupies the inner [±45]_2_ orientation (3573 N). Similarly, in the three-point bending tests, the C45 tubes exhibited the highest bending resistance, with a peak force of 5161 N (flexural strength 92.8 MPa) and an elastic stiffness of 742 N/mm. Notably, despite its lower peak force, the G45 configuration absorbed a comparable total energy and specific energy absorption, reflecting its superior damage tolerance. However, when evaluated in terms of statistical analyses and damage tolerance, the very low coefficients of variation exhibited by the G45 configuration revealed that this structure is much more predictable in terms of deformation stability. Scanning Electron Microscopy (SEM) examinations confirmed these macroscopic data at the microstructural level; while brittle fractures in the form of sudden fiber fractures and severe delaminations were observed in the C45 specimens, more energy-dissipating ductile damage mechanisms, such as matrix crushing, were detected in the G45 specimens due to the high elongation capability of glass fiber. In light of the obtained findings, it was concluded that the C45 stacking sequence is preferable in components requiring absolute stiffness and high load-bearing capacity in industrial lightweight structural designs, whereas the G45 is preferable in applications where energy absorption capacity, damage tolerance, and structural ductility are critical.

## Figures and Tables

**Table 1:** Mechanical properties of the constituent fibers and the epoxy matrix.

Constituent	σ (MPa)	ρ (g/cm^3^)	E (GPa)	ε Break (%)
Carbon 12K A-42	3500	1.78	230	1.5
E-glass 600 tex	2200	2.54	70	1.5–2
Epoxy Mixture	50–60	1.2	3.4	4–6

**Table 2:** Properties and fiber volume fractions of the hybrid tube configurations.

Configuration	Density (g/cm^3^)	Inner Layer	Outer Layer	Vf	Thickness (mm)	Tube Length (mm)
Config Carbon 45-1	1.84	[±45]_2_ Carbon	[±75]_2_ Glass	0.61	3.03	36 and 140
Config Carbon 45-2	1.70	[±45]_2_ Carbon	[±75]_2_ Glass	0.54	3.53	36 and 140
Config Carbon 45-3	1.71	[±45]_2_ Carbon	[±75]_2_ Glass	0.53	3.39	36 and 140
Config Carbon 45-4	1.73	[±45]_2_ Carbon	[±75]_2_ Glass	0.54	3.41	36 and 140
Config Carbon 45-5	1.68	[±45]_2_ Carbon	[±75]_2_ Glass	0.57	3.24	36 and 140
Config Glass 45-1	1.73	[±45]_2_ Glass	[±75]_2_ Carbon	0.53	3.00	36 and 140
Config Glass 45-2	1.80	[±45]_2_ Glass	[±75]_2_ Carbon	0.59	2.61	36 and 140
Config Glass 45-3	1.75	[±45]_2_ Glass	[±75]_2_ Carbon	0.58	3.00	36 and 140
Config Glass 45-4	1.77	[±45]_2_ Glass	[±75]_2_ Carbon	0.55	3.12	36 and 140
Config Glass 45-5	1.74	[±45]_2_ Glass	[±75]_2_ Carbon	0.57	3.08	36 and 140

**Table 3:** Energy absorption indices for the specimens under quasi-static radial compression (n = 5, integration over 0–13 mm).

Configuration	Mass (g)	Max. Force (N)	E (J)	SEA (J/g)	δ (mm)
C45 (n = 5)	14.99 ± 0.55	4209 ± 322	39.50 ± 1.36	2.64 ± 0.13	13.0
COV (%)	3.64	7.64	3.45	4.77	-
G45 (n = 5)	15.21 ± 0.24	3573 ± 136	31.34 ± 0.34	2.06 ± 0.03	13.0
COV (%)	1.58	3.82	1.08	1.64	-

**Table 4:** Energy absorption indices for the specimens under quasi-static three-point bending (n = 5, integration over 0–20 mm).

Configuration	Mass (g)	Max. Force (N)	E (J)	P_m_ (kN)	SEA (J/g)	δ (mm)
C45 (n = 5)	58.28 ± 2.12	5161 ± 249	62.88 ± 6.26	3.14 ± 0.31	1.08 ± 0.14	20.0
COV (%)	3.64	4.83	9.95	9.95	13.01	-
G45 (n = 5)	59.15 ± 0.93	4731 ± 164	66.96 ± 2.83	3.35 ± 0.14	1.13 ± 0.06	20.0
COV (%)	1.58	3.47	4.22	5.12	5.12	-

**Table 5:** Derived lamina elastic constants of the carbon/epoxy and glass/epoxy layers (V_f_ = 0.56).

Lamina	E_1_ (GPa)	E_2_ (GPa)	G_12_ (GPa)	ν_12_
Carbon/epoxy	130.3	7.73	3.52	0.266
Glass/epoxy	40.7	13.03	3.91	0.277

**Table 6:** Lamina strength properties used in the Tsai–Wu evaluation (MPa).

Lamina	X_t_	X_c_	Y_t_	Y_c_	S
Carbon/epoxy	1984	1290	50	200	70
Glass/epoxy	1256	817	51	204	78

X_t_, X_c_: longitudinal tensile and compressive strengths; Y_t_ and Y_c_: transverse tensile and compressive strengths; S: in-plane shear strength.

**Table 7:** Tsai–Wu first-ply failure prediction for the inner [±45]_2_ layer of each configuration.

Configuration (İnner Layer)	Strength Ratio (SR)	Damage İnitiation
C45 inner [±45]_2_ carbon	0.82	earlier
G45 inner [±45]_2_ glass	0.95	later

A lower strength ratio indicates that the inner layer reaches the Tsai–Wu failure envelope at a lower load, i.e., earlier damage initiation. The values are used for relative comparison between the two configurations.

## Data Availability

The original contributions presented in this study are included in the article. Further inquiries can be directed to the corresponding author.

## Abbreviations

The following abbreviations are used in this manuscript:

ASTM	American Society for Testing and Materials
CFRP	Carbon Fiber-Reinforced Polymer
CV	Coefficient of Variation
FW	Filament Winding
GFRP	Glass Fiber-Reinforced Polymer
PRD	Percent Ring Deflection
SEA	Specific Energy Absorption
SEM	Scanning Electron Microscopy
SR	Strength Ratio
Vf	Fiber Volume Fraction
